# Hypofractionated Radiotherapy in African Cancer Centers

**DOI:** 10.3389/fonc.2020.618641

**Published:** 2021-02-19

**Authors:** William Swanson, Francesca Kamwa, Richard Samba, Taofeeq Ige, Nwamaka Lasebikan, Abba Mallum, Twalib Ngoma, Erno Sajo, Ahmed Elzawawy, Luca Incrocci, Wilfred Ngwa

**Affiliations:** ^1^ Department of Physics and Applied Physics, University of Massachusetts Lowell, Lowell, MA, United States; ^2^ Department of Radiation Oncology, Dana Farber Cancer Institute, Boston, MA, United States; ^3^ Department of Regulation and Regulatory Control, Cameroon National Radiation Protection Agency, Yaounde, Cameroon; ^4^ Department of Medical Physics, National Hospital, Abuja, Nigeria; ^5^ Department of Medical Physics, University of Abuja, Abuja, Nigeria; ^6^ Department of Radiation Medicine, University of Nigeria-Teaching Hospital, Enugu, Nigeria; ^7^ Department of Oncology, Inkosi Albert Luthuli Central Hospital, Durban, South Africa; ^8^ Department of Radiology/Radiotherapy, Muhimbili University of Health and Allied Sciences, Dar es Salaam, Tanzania; ^9^ Department of Clinical Oncology, Suez Canal University, Ismailia, Egypt; ^10^ Department of Radiotherapy, Erasmus MC, Rotterdam, Netherlands; ^11^ Department of Radiation Oncology, Brigham and Women’s Hospital, Boston, MA, United States; ^12^ Department of Radiation Oncology, Harvard Medical School, Boston, MA, United States

**Keywords:** hypofractionation, radiotherapy, Africa, radiation, cancer

## Abstract

In the advent of the coronavirus disease (COVID-19) pandemic, professional societies including the American Society for Radiation Oncology and the National Comprehensive Cancer Network recommended adopting evidence-based hypofractionated radiotherapy (HFRT). HFRT benefits include reduction in the number of clinical visits for each patient, minimizing potential exposure, and reducing stress on the limited workforce, especially in resource-limited settings as in Low-and-Middle-Income Countries (LMICs). Recent studies for LMICs in Africa have also shown that adopting HFRT can lead to significant cost reductions and increased access to radiotherapy. We assessed the readiness of 18 clinics in African LMICs to adopting HFRT. An IRB-approved survey was conducted at 18 RT clinics across 8 African countries. The survey requested information regarding the clinic’s existing equipment and human infrastructure and current practices. Amongst the surveyed clinics, all reported to already practicing HFRT, but only 44% of participating clinics reported adopting HFRT as a common practice. Additionally, most participating clinical staff reported to have received formal training appropriate for their role. However, the survey data on treatment planning and other experience with contouring highlighted need for additional training for radiation oncologists. Although the surveyed clinics in African LMICs are familiar with HFRT, there is need for additional investment in infrastructure and training as well as better education of oncology leaders on the benefits of increased adoption of evidence-based HFRT during and beyond the COVID-19 era.

## Introduction

The novel coronavirus disease 2019 (COVID-19) pandemic hit Africa’s healthcare infrastructure, already struggling to address a growing burden of cancer with over 1 million cases and 700,000 deaths per year and with major disparities in access to care (1). COVID-19 restrictions resulted in limited inpatient access including limitations in patients travelling to receive treatment and reduction in financial resources to access care (2, 3). In terms of cancer radiotherapy (RT), treatments have been delayed due to reduced staff and competition for resources such as computed tomography (CT) imaging. The American Society for Radiation Oncology (ASTRO) and the National Comprehensive Cancer Network (NCCN) issued recommendations on greater adoption of evidence-based hypofractionated RT (HFRT), where currently expanding evidence supports effective HFRT treatment outcomes (4–6). The recommendations were designed to support safe and effective RT in a way that could relieve stress on reduced resources and reduce COVID-19 exposure risks to patients and staff (7).

In perspective, cancer management and control in Low-and-Middle-Income Countries (LMICs) remains a challenge due to critical shortages of qualified healthcare professionals and clinical infrastructure. The COVID-19 pandemic further exposed these challenges and already existing disparities in access to radiotherapy. HFRT, where fewer radiotherapy fractions are employed compared to conventional fractionated RT (CFRT), presents opportunity to alleviate these challenges. HFRT permits reducing the number of visits a patient makes to the clinic for radiotherapy, reducing stress on staff as well as costs, and enabling clinics to treat more patients in less time. Recent work has highlighted the potential to significantly increase access to care for patients in Africa with wider adoption of HFRT for prostate and breast cancer (8), which are amongst the leading causes of cancer deaths (9, 10). Through clinical trials, HFRT has demonstrated non-inferior outcomes compared to CFRT for breast and prostate cancer (11, 12). The 2002–2011 CHHiP prostate HFRT clinical trial resulted in outcomes with equivalent or increased 5-year relapse-free survival rates (RFS) when delivering 60 Gy over 20 fractions (3 Gy/fraction) compared to CFRT with 74 Gy in 37 fractions (2 Gy/fraction) (11, 13). The 2007-2010 HYPRO prostate HFRT clinical trial also reported equivalent 5-year RFS when delivering 64.6 Gy over 19 fractions (3.4 Gy/fraction) compared to CFRT with 78 Gy over 39 fractions (2 Gy/fraction) at the cost of limited increased risk of acute side-effects (11, 14). Similarly, several breast HFRT clinical trials demonstrated equivalent outcomes when treating with various HFRT schedules, 2.7–3.3 Gy/fraction, versus the CFRT schedule of 2 Gy/fraction (50 Gy in 25 fractions) (12, 15, 16). In addition to equivalent clinical outcomes, patients consider HFRT significantly more convenient besides the other benefits highlighted above.

Because HFRT involves increased doses per fraction, quality assurance (QA) of treatment planning and delivery is even more important to maintain. RT Machines and equipment must maintain performance quality within tolerance and high-precision patient alignment and motion management are necessary. Personnel must have adequate training to perform QA, treatment planning, and treatment delivery. Whether CFRT or HFRT, it is crucial that any clinic has the level of infrastructure to perform RT safely and effectively. Although some LMIC clinic infrastructure data is available from the International Atomic Energy Agency (IAEA) Directory of Radiotherapy Centres (DIRAC), there are no reports regarding clinical ability to perform HFRT. Here, we assess the “readiness” of African LMIC clinics in adopting evidence-based HFRT techniques recommended and made even more urgent by COVID-19.

## Method

In this study, we define the readiness of a clinic in adopting curative HFRT by several criterion. As HFRT is similar in principle to stereotactic body RT (SBRT) by delivering fewer higher-dose fractions to an often-times immobilized patient, the readiness criteria was conservatively based on the SBRT recommendations provided in the American Association of Physicists in Medicine Task Group Report 101 (TG101) (17). TG101 provides recommendations with regard to the type of RT machine used, image guiding and motion management techniques, and minimum necessary quality insurance parameters in order to deliver SBRT safely and effectively. These same recommendations, although very conservative, can be applied to HFRT to accommodate the increased dose rate. Readiness criteria we deemed critical as per TG101 recommendations included the following: a) An RT machine capable of millimeter precision, b) An on-board CT system or an electronic portal imaging device, c) both forward and inverse treatment planning systems, d) a combination of patient immobilization tools, e) a motion tracking system, and f) sufficient personnel training. Case-dependent, HFRT may need higher precision and expertise than CFRT, requiring more sophisticated equipment and expertise.

We conducted an online survey, approved by the University of Massachusetts Lowell Institutional Review Board, to assess clinical HFRT practice, available RT infrastructure, and interest in HFRT. The online survey was distributed to healthcare administrators in African RT clinics through personal contacts, inviting RT staff participation. The survey was accessible *via* a Google Form over a 3-month period. Participants included 34 RT health professionals from 18 RT clinics across 8 African countries (**Supplementary Figure 1**). Survey responses varied between 1 and 4 participants per clinic, including radiation oncologists, medical physicists, dosimetrists, quality assurers, and radiation therapists. Note that many of these participants assume more than one of the listed roles in their clinics.

Survey items requested participants to report various demographic information, current practices and infrastructure in their clinic, and their clinical role and level of training. Clinic infrastructure questions included the types of RT machines, imaging and treatment planning methods, image-guidance methods, and motion management tools. Reporting a lack of specialized equipment may indicate a lack of readiness to adopt HFRT. Current practices of the surveyed clinics were reported as their frequency of use of HFRT, which cancers they typically treated with HFRT, and what fractionated doses they typically used in RT delivery in general. The practiced dose rate reports would suggest if they are delivering appropriate dose rates for CFRT/HFRT or if they are safely capable of delivering even greater stereotactic body RT levels of dose. Lastly, participants were requested to self-report if they received sufficient training in the following subjects: Radiation Safety, RT Delivery, Patient Immobilization and Motion Management, Treatment Planning, and RT Quality Assurance. Insufficient training reports would suggest a non-ideal situation to introduce new RT methods. These items were selected to determine if clinics possess the minimum level of equipment, experience, and training necessary to perform HFRT safely. The survey items are included in the **Supplementary Material**.

## Results

**Figure 1:**
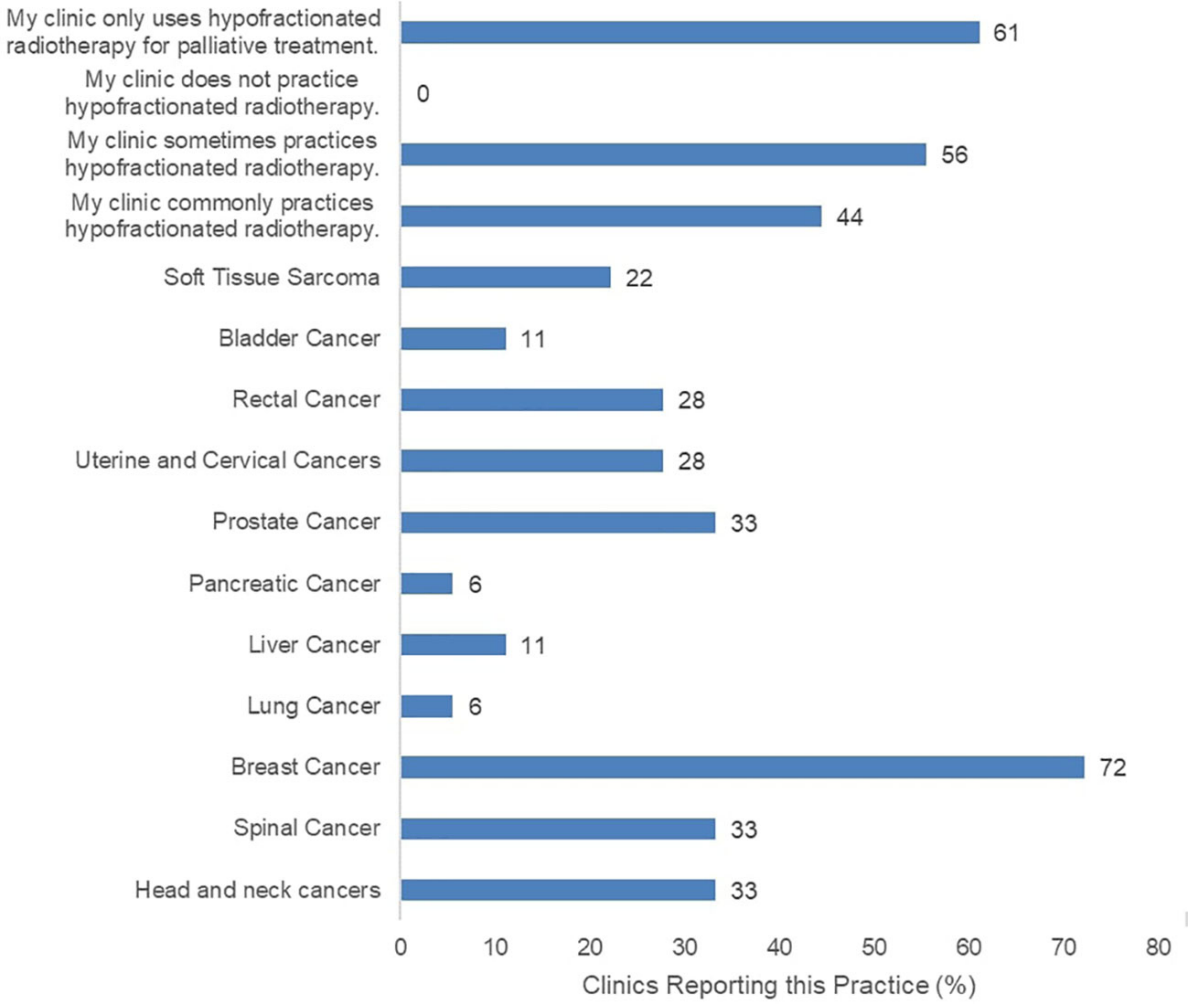
Reported HFRT Practices including potential treatment targets across 18 surveyed clinics.

The surveyed clinics reported current practice of HFRT for various indications (**Figure 1**). The most common HFRT-treated patients were those with breast cancer at 72%, and prostate cancer at 33%. Eight of the 18 participating clinics reported infrequent practice of HFRT while another 8 clinics reported common practice of HFRT. However, 11 of all participating clinics report that HFRT is only practiced for palliative care, not curative treatment. Clinics were also asked to report typical prescription doses for confirmation of HFRT fractionated dose rate practice (**Supplementary Figure 2**). Most clinics confirmed to deliver varying low and high prescription dose rates, including typical CFRT doses (2 Gy or below per fraction), and HFRT doses (between 2 Gy and 5 Gy per fraction). Meanwhile, 67% of clinics reported use of RT with fraction size of 5 Gy or greater.

**Figure 2:**
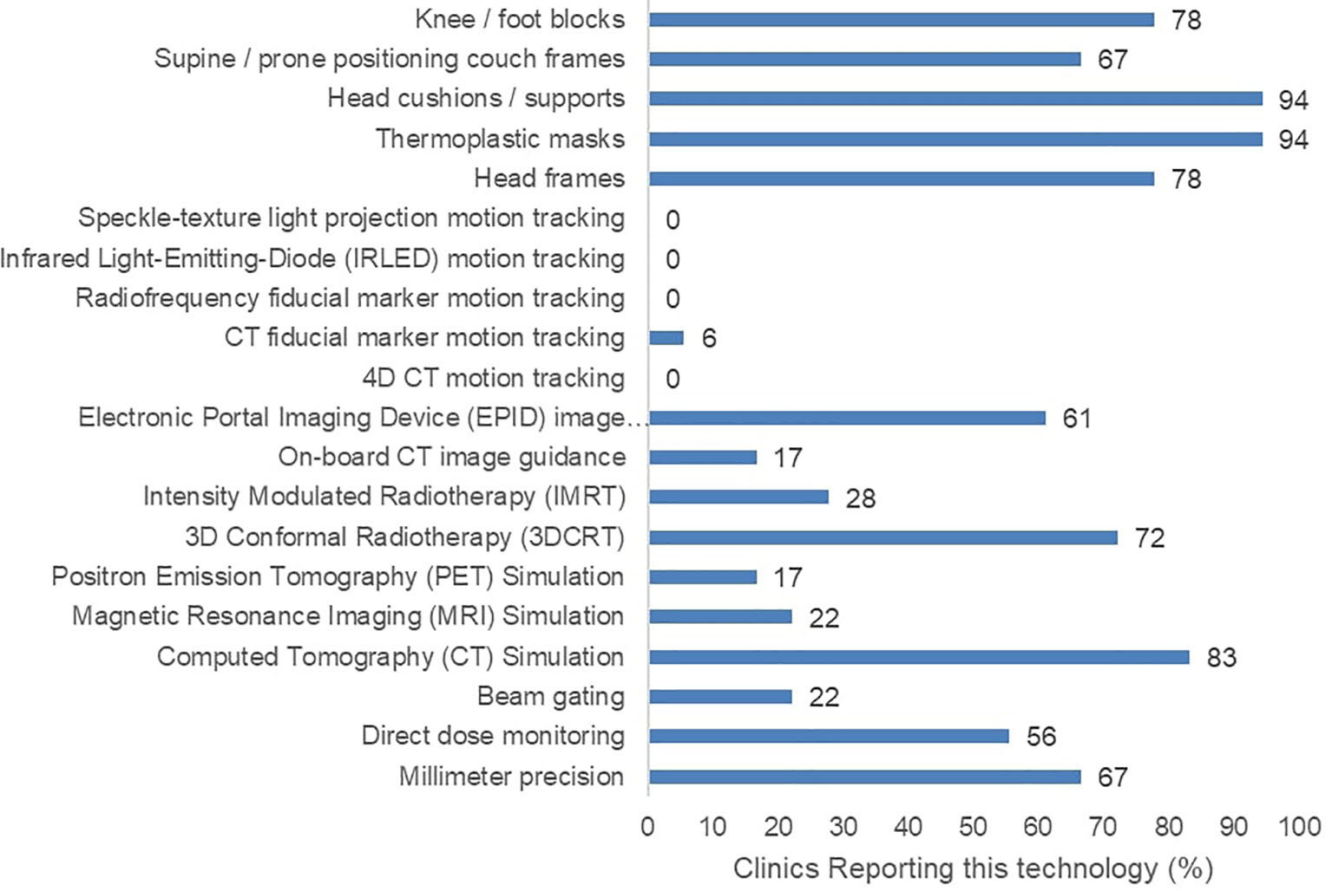
Reported radiotherapy technology including treatment machine features, image guidance, and patient immobilizing apparatuses across 18 surveyed clinics.

Furthermore, 17 of the 18 surveyed clinics had at least one external-beam RT (EBRT) machine. However, they reported varying degrees of precision, image-guidance options, and motion management options (**Figure 2**). None of the participating clinics reported the ability to perform advanced motion tracking image guidance techniques involving speckle-texture projection, infrared LEDs, or 4D computed tomography. Only a single participating clinic confirmed the use of radiopaque fiducial markers in any RT practice. Regarding treatment strategies, five participating clinics reported the use of intensity modulated RT (IMRT). Lastly, five participating clinics reported an absence of CT treatment simulation in their facilities.

Those surveyed were also asked if they received specific training experiences from a certified professional, including radiation safety, dosimetry and treatment planning, QA methods, treatment delivery, and motion management techniques (**Supplementary Figure 3**). The training report showed most staff reporting the appropriate training for their associated roles. All participating radiation therapists indicated that they have received sufficient training in RT delivery and motion management. All participating dosimetrists and medical physicists indicated to have received sufficient training in treatment planning and quality assurance. Only one therapist and physicist reported the lack of adequate training in radiation safety. Twelve of the 16 participating radiation oncologists indicated to have received adequate treatment planning training, suggesting additional training may be needed for contouring according to their available infrastructure and common practices.

## Discussion

Radiotherapy resources are highly limited in Africa, in which 29 out of the 56 countries do not have radiotherapy units (18). The large patient burden on these few clinics makes the need for quality assurance ever more critical. The present study surveyed 18 clinics and the results suggest that many of them fulfilled some but not all the readiness criteria based on the information received regarding the availability of sufficient RT infrastructure and received training. Regarding infrastructure, the little-to-no motion tracking equipment present in the participating clinics is concerning. Without motion tracking systems, the increased fraction dose in HFRT can be dangerous if not properly delivered, especially for cases of breast or lung cancer where tumor motion is significant. Regarding the participating clinics’ RT practices, it is important to note that many report having experience in delivering both HFRT and SBRT level fraction doses. However, the reported level of equipment suggests that the high-dose fractions may be unsafe to deliver. The reported training received by the participants demonstrated moderate competency in the appropriate roles with some exceptions. Several participating dosimetrists, physicists, and oncologists reported insufficient training in immobilization and motion management. This aligns with the reported lack of motion tracking systems in the clinics. Additionally, the participating oncologists reported a lack of training in radiation safety, motion management, and QA. This may be due to compartmentalizing of clinical roles. The lack of motion management training among many participants suggests that the clinical staff may not be prepared to deliver HFRT where these considerations are needed.

Therefore, additional investment in infrastructure and training are needed in such area so that HFRT can be performed safely and effectively. This agrees with a previous study which highlighted the importance of suitable equipment, well-trained personnel, and other radiotherapy support systems for effective application of HFRT (8). These clinics can act in the short term by implementing additional training for staff in areas of RT motion management. This continued education can be applied to current systems for greater treatment precision and improved QA. Curative HFRT could potentially be performed safely in these clinics for less severe cases where motion tracking is not critical. However, long term infrastructure investment would be required to perform curative HFRT for more severe cases where motion tracking and superior image guidance would be necessary. To adopt HFRT for more complex cases, high precision RT machines, image guidance devices, and motion tracking solutions including 4D CT and fiducial marker tracking would be needed.

A limitation of this study is that the data originates from only 18 centers among the RT clinics across Africa. So this data from a relatively small sample may not reflect the situation in other centers. Despite this limitation, the results in this study provide a representative snapshot of the capacity for African cancer centers to adopt HFRT where many African countries lack the ability to deliver RT altogether. More studies are needed to assess readiness for each country in Africa with RT machines. Another limitation is that the self-reported data regarding the current practices and received training is inherently subjective. It is not clear if the current palliative or curative HFRT practices in these clinics are performed to acceptable quality standards nor is it clear if the level of training is uniform across different countries. Patient level treatment quality investigation would be necessary to verify RT performance quality. This highlights the need to establish more uniform standards in assessing the level of competence of radiation oncology health professionals. Additional research capacity must also be strengthened to avoid lack of data or under-reported information regarding cancer in the Africa continent as whole.

Many African cancer centers already employ HFRT, with the majority using this for palliative care. Some countries are already reporting increase in adoption during the COVID-19 era. However, in some centers, additional investments in infrastructure and training may be needed to allow recommended increased adoption of HFRT with adequate safety, which can substantially increase access to care during the COVID-19 pandemic and beyond.

## Data Availability Statement

The datasets may not be shared due to identifiable ties to individuals and organizations. Requests to access the datasets should be directed to WS, William_Swanson@student.uml.edu.

## Ethics Statement

The studies involving human participants were reviewed and approved by The University of Massachusetts Lowell Institutional Review Board. The patients/participants provided their written informed consent to participate in this study.

## Author Contributions

Study design was contributed by WS, FK, RS, TI, NL, AM, TN, AE, LI, WN. Data acquisition was contributed by WS, FK, RS, TI, NL, AM, TN, AE, LI, WN. Data analysis was contributed by WS, FK, RS, AE, LI, WN. Manuscript writing was contributed by WS, FK, RS, ES, LI, WN. All authors contributed to the article and approved the submitted version.

## Funding

This work is funded by the Brigham Research Institute. Research reported in this publication was partially supported by the National Institutes of Health under Award Number R01CA239042. The content is solely the responsibility of the authors and does not necessarily represent the views of the National Institutes of Health.

## Conflict of Interest

The authors declare that the research was conducted in the absence of any commercial or financial relationships that could be construed as a potential conflict of interest.
