# Supplementary material for: Hypofractionated Radiotherapy in African Cancer Centers
Source: Front Oncol. 2021 Feb 19;10:618641. doi: 10.3389/fonc.2020.618641 (PMC7933544; doi:10.3389/fonc.2020.618641)
Supplement: Supplementary file 1 [file DataSheet_1.docx]

**Supplementary Material**


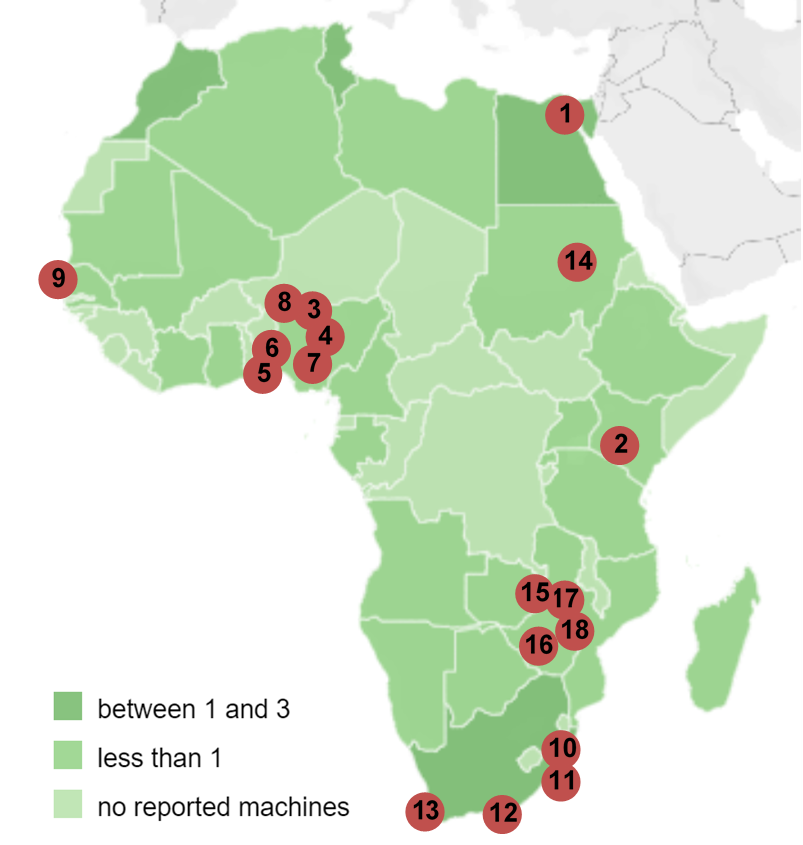


**Supplementary Figure 1.** RT machines in Africa per million people as of March 2020.^17^ The countries indicated in the lightest green report zero RT machines where the countries indicated in the darkest green report between 1-3 RT machines per million people. Surveyed clinics are labeled as noted with the number of responding representatives: (1) National Cancer Institute, Egypt, 2; (2) Kenyatta National Hospital, Kenya, 1; (3) Ahmadu Bello University Teaching Hospital Zaria, Nigeria, 1; (4) National Hospital Abuja, Nigeria, 2; (5) NSIA-LUTH Cancer Center, Nigeria, 4; (6) University College Hospital Ibadan, Nigeria, 4; (7) University of Nigeria Teaching Hospital Enugu, Nigeria, 3; (8) Usmanu Danfodiyo University Teaching Hospital Sokoto, Nigeria, 1; (9) Hospital Aristide Le Dantec, Senegal, 1; (10) Addington Hospital, South Africa, 1; (11) Albert Luthuli Central Hospital, South Africa, 4; (12) Livingstone Tertiary Hospital, South Africa, 1; (13) Tygerberg Hospital, South Africa, 1; (14) National Cancer Institute, Sudan, 1; (15) Cancer Diseases Hospital, Zambia, 1; (16) Mpilo Central Hospital, Zimbabwe, 1; (17) Oncocare, Zimbabwe, 1; (18) Parirenyatwa Radiotherapy Centre, Zimbabwe, 3.

**
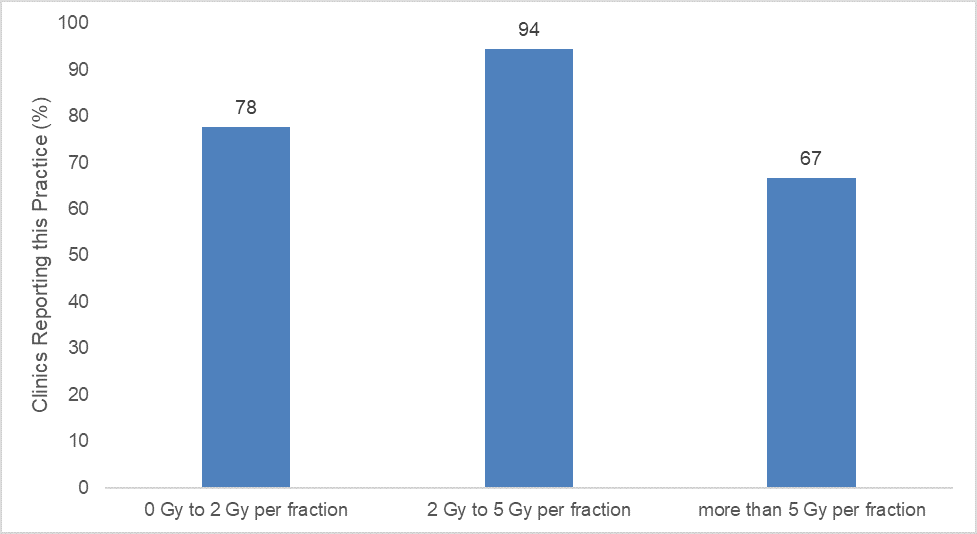
**

**Supplementary Figure 2.** Reported typical RT prescription doses in dose per fraction.

**
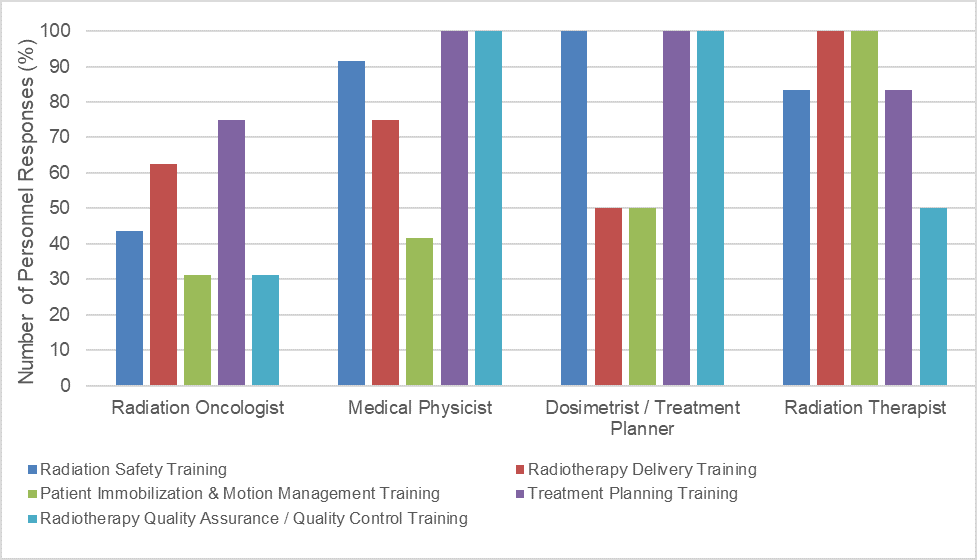
Supplementary Figure 3.** Reported training received by participating staff sorted by clinical role.

Below is the survey as presented to participants.

**HFRT Access Survey**

In the face of the novel coronavirus (COVID-19) pandemic, recognizing the potential for staff reductions, professional societies have recommended that radiation oncology health professionals continue to follow appropriate evidence-based guidelines (e.g., National Comprehensive Cancer Network guidelines) while striving for the shortest possible course of radiotherapy e.g. single-fraction treatment for bone pain, or hypofractionation where appropriate (e.g., breast, prostate). This recommendation will also reduce the number of times patients have to travel to cancer centers under COVID-19 restrictions and minimize their exposure to the virus. This survey seeks to access the gaps in infrastructure and knowledge that would need to be addressed to safely implement hypofractionation in Africa.

All responses will remain confidential.

Estimated completion time: 7 minutes

By submitting the survey, you consent to the use of this data for academic research.

Email Address: __________

1. Please provide the name of your clinic.
2. In which country is your clinic located?
3. What is your position in the clinic? (select all that apply)
   1. Radiation Oncologist
   2. Medical Physicist
   3. Dosimetrist / Treatment Planner
   4. Quality Assurance / Quality Control
   5. Radiation Therapist
   6. Nurse
   7. Administrator
   8. Regulator
4. Indicate the training you received by a certified professional. (select all that apply)
   1. Radiation Safety Training
   2. Radiotherapy Delivery Training
   3. Patient Immobilization & Motion Management Training
   4. Treatment Planning Training
   5. Radiotherapy Quality Assurance / Quality Control Training
   6. None of the Above
5. How many megavoltage linear accelerators (LINACs) are active in your clinic?
6. How many cobalt-60 machines are active in your clinic?
7. How many orthovoltage machines are active in your clinic?
8. Which features are your radiotherapy machines capable of performing? (select all that apply)
   1. Millimeter precision
   2. Direct dose monitoring
   3. Beam gating
   4. None of the Above
9. Which imaging modalities and treatment planning systems do you have available for treatment simulation, treatment planning, and motion tracking/management? (select all that apply)
   1. Computed Tomography (CT) Simulation
   2. Magnetic Resonance Imaging (MRI) Simulation
   3. Positron Emission Tomography (PET) Simulation
   4. 3D Conformal Radiotherapy (3DCRT)
   5. Intensity Modulated Radiotherapy (IMRT)
   6. Volumetric Modulated Arc Therapy (VMAT)
   7. On-board CT image guidance
   8. Electronic Portal Imaging Device (EPID) image guidance
   9. 4D CT motion tracking
   10. CT fiducial marker motion tracking
   11. Radiofrequency fiducial marker motion tracking
   12. Infrared Light-Emitting-Diode (IRLED) motion tracking
   13. Speckle-texture light projection motion tracking
   14. None of the Above
10. Which of the following apparatuses does your clinic have available for patient immobilization? (select all that apply)
    1. Head frames
    2. Thermoplastic masks
    3. Head cushions / supports
    4. Supine / prone positioning couch frames
    5. Knee / foot blocks
    6. None of the Above
11. What prescription doses (excluding boost treatment) does your clinic commonly deliver in external-beam radiotherapy? (select all that apply)
    1. 0 Gy to 2 Gy per fraction
    2. 2 Gy to 5 Gy per fraction
    3. more than 5 Gy per fraction
12. If applicable, which cancer sites does your clinic perform curative treatment using hypofractionated radiotherapy? (select all that apply)
    1. My clinic does not practice hypofractionated radiotherapy.
    2. My clinic only uses hypofractionated radiotherapy for palliative treatment.
    3. Head and neck cancers
    4. Spinal Cancer
    5. Breast Cancer
    6. Lung Cancer
    7. Liver Cancer
    8. Pancreatic Cancer
    9. Prostate Cancer
    10. Uterine and Cervical Cancers
    11. Rectal Cancer
    12. Bladder Cancer
    13. Soft Tissue Sarcoma
13. Describe your familiarity with hypofractionated radiotherapy?
    1. My clinic commonly practices hypofractionated radiotherapy.
    2. My clinic sometimes practices hypofractionated radiotherapy.
    3. I am familiar with hypofractionated radiotherapy.
    4. I am NOT familiar with hypofractionated radiotherapy and I am interested in learning more.
    5. I am NOT familiar with hypofractionated radiotherapy.
14. Please provide any additional information to clarify any answers above, or that you deem important to share regarding hypofractionated radiotherapy.
